# Achieving High Accuracy Prediction of Minimotifs

**DOI:** 10.1371/journal.pone.0045589

**Published:** 2012-09-27

**Authors:** Tian Mi, Sanguthevar Rajasekaran, Jerlin Camilus Merlin, Michael Gryk, Martin R. Schiller

**Affiliations:** 1 Department of Computer Science and Engineering, University of Connecticut, Storrs, Connecticut, United States of America; 2 Department of Molecular, Microbial, and Structural Biology, University of Connecticut Health Center, Farmington, Connecticut, United States of America; 3 School of Life Sciences, University of Nevada Las Vegas, Las Vegas, Nevada, United States of America; Indian Institute of Science, India

## Abstract

The low complexity of minimotif patterns results in a high false-positive prediction rate, hampering protein function prediction. A multi-filter algorithm, trained and tested on a linear regression model, support vector machine model, and neural network model, using a large dataset of verified minimotifs, vastly improves minimotif prediction accuracy while generating few false positives. An optimal threshold for the best accuracy reaches an overall accuracy above 90%, while a stringent threshold for the best specificity generates less than 1% false positives or even no false positives and still produces more than 90% true positives for the linear regression and neural network models. The minimotif multi-filter with its excellent accuracy represents the state-of-the-art in minimotif prediction and is expected to be very useful to biologists investigating protein function and how missense mutations cause disease.

## Introduction

Minimotifs (also called Short Linear Motifs) are short contiguous peptide pieces of proteins that have a known biological function, which can be categorized into binding, posttranslational modification of the minimotif, and protein trafficking. Minimotifs are involved in nearly all cell processes including intracellular signaling, extra-cellular activities, and disease [1]–[4].

Minimotifs contain both a known biological function and a short protein sequence representation generally of less than 15 amino acids which distinguishes them from protein domains like those in ProSite and other tools such as MEME and SCOP that identify sequence patterns, but do not have known functions [5], [6]. Computational minimotif prediction tools have arisen to perform searches and predict new functions in proteins based upon established functions associated with minimotifs in other proteins. Minimotif Miner (MnM), Eukaryotic Linear Motif (ELM), and ScanSite fulfill these roles [3], [7], [8]–[11]. These approaches do have value in their successes; however, the relatively low sequence complexity of minimotifs gives rise to many false positives, which limit their usefulness.

Our approach to this problem has developed five separate scores/filters each of which has a significant value in reducing false positive predictions [7], [8], [12], [13]. Frequency Score analysis (FS) uses the complexity of minimotif sequence definitions to rank-order minimotifs. A Surface Prediction (SP) algorithm identifies minimotifs likely to be on the surface of a protein. The remaining three approaches take advantage of both the target and source proteins. The Protein-Protein Interaction filter (PPI) refines minimotif predictions by selecting only motifs whose source protein and target protein are known to interact in vivo, eliminating any whose source protein and target protein do not interact [12]. In addition to exact PPIs, protein-protein interactions are also expanded based on orthologues and paralogues across species and taxa (“HomoloGene-PPI”), as well as sequence similarity (“Similarity-PPI”). The Cellular or Molecular Function filters (CF/MF), retain minimotifs whose source protein and target protein share a common cellular or molecular function, respectively [13]. Exact functional matching is not required; rather function terms are related through the network structure provided by the Gene Ontology (GO) database [14]. For example, one function may be a subclass of another function, or one function may regulate another function.

These scores/filters exploit different components of a minimotif syntax developed for this purpose [15]. We next demonstrated that pairwise combinations of filters were better than either alone, suggesting that each filter used distinct information. This led us to perform a systematic comparison of different combinations of five scores/filters that we had developed previously. A study of minimotif filtering with linear regression, support vector machine, and neural network algorithms shows a vast improvement in minimotif prediction with accuracies above 85% and in one analysis less than 1% false positives while retaining more than ∼90% of the true positives. This advance sets us on a path to vastly reducing false positive predictions. Implementation of this filter combination on the MnM website renders minimotif-mediated protein function prediction much more reliable and influential.

## Results

To build and test the multi-filter approach we used five existing filters designed to remove false-positive minimotifs [7], [8], [12], [13]. This multi-filter approach was enabled in large part due to a rich model of the syntactical and semantic structure for minimotifs [14]. Briefly, a minimotif is found in a ‘source protein’ and the target protein binds the minimotif or alters the minimotif. Two of the filters are based upon regular expression searches involving solely the source protein (where the minimotif is found). Frequency Score analysis (FS) uses the complexity of minimotif sequence definitions to rank-order minimotifs. A surface prediction algorithm identifies minimotifs likely to be on the surface of a protein.

**Figure 1:**
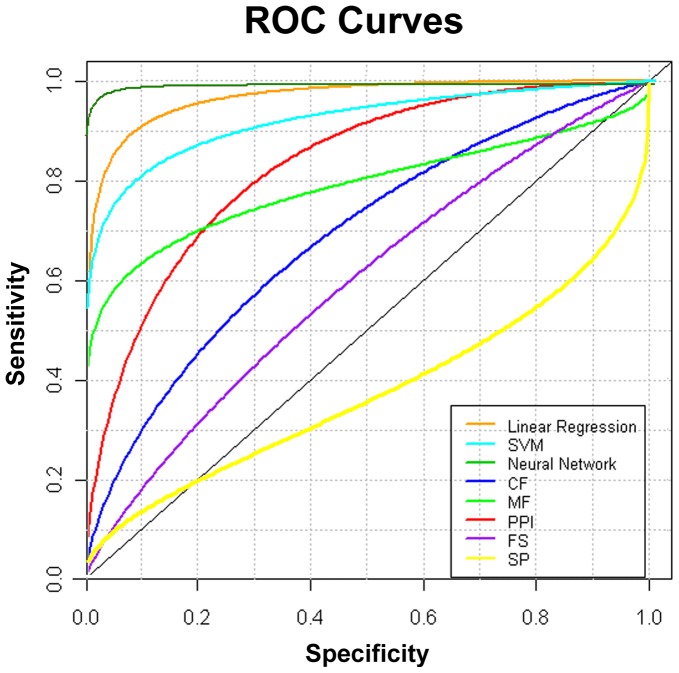
ROC plots comparing linear regression, support vector machine, and neural network multi-filters with, individual CF, MF, PPI, FS, and SP filters. ROCs are colored orange for linear regression, cyan for support vector machine, cyan dark green for neural network, red for PPI filter, blue for CF filter, green for MF filter, purple for FS filter, and yellow for SP filter.

We first evaluated each individual filter on the same dataset by generating Receiver Operator Curves (ROCs) and comparing the area under the curves (AUC) (**Fig. 1** **,** **Table 1**). The (AUC) for individual filters ranged from 0.72–0.88, indicating good filter performance. There is much room for improvement.

**Table 1:** ROC statistics for individual motif filters.

Method	AUC	P-value
CF	0.72	0.12
MF	0.83	0.03
FS	0.72	0.08
PPI	0.88	1.4×10^−3^
SP	0.38	1.00

We evaluated several approaches for combining differing filtering techniques. Linear regression, support vector machine, and neural network multi-filter models were trained and tested by randomly partitioning the true positive and true negative data equally into five groups, each of which contained a subset of 400 instances. A five-fold cross validation was performed by successively using four groups to train the multi-filter models and one group of validation data to evaluate the effectiveness of the multi-filter. The three multi-filter models used the individual CF, MF, FS, PPI, and SP minimotifs filters. The AUC values indicated that the multi-filters were significantly better than any individual filter (**Table 2**).

**Table 2:** ROC statistics for three minimotif multi-filter models.

5-fold cross validation		#1	#2	#3	#4	#5	Average	Standard Deviation
linear regression	AUC	96.4%	96.7%	96.6%	96.2%	95.9%	96.7%	0.3%
	P-Value	2.3*10^−114^	5.3*10^−116^	1.3*10^−115^	1.9*10^−113^	4.2*10^−112^	–	–
support vector machine	AUC	93.6%	92.9%	93.8%	92.9%	93.8%	93.4%	0.5%
	P-Value	9.8*10^−259^	1.2*10^−178^	3.3*10^−266^	<10^−325^	1.7*10^−266^	–	–
neural network	AUC	99.6%	99.0%	99.8%	99.3%	96.7%	98.9%	1.3%
	P-Value	<10^−325^	<10^−325^	<10^−325^	<10^−325^	1.7*10^−211^	–	–

**Figure 2:**
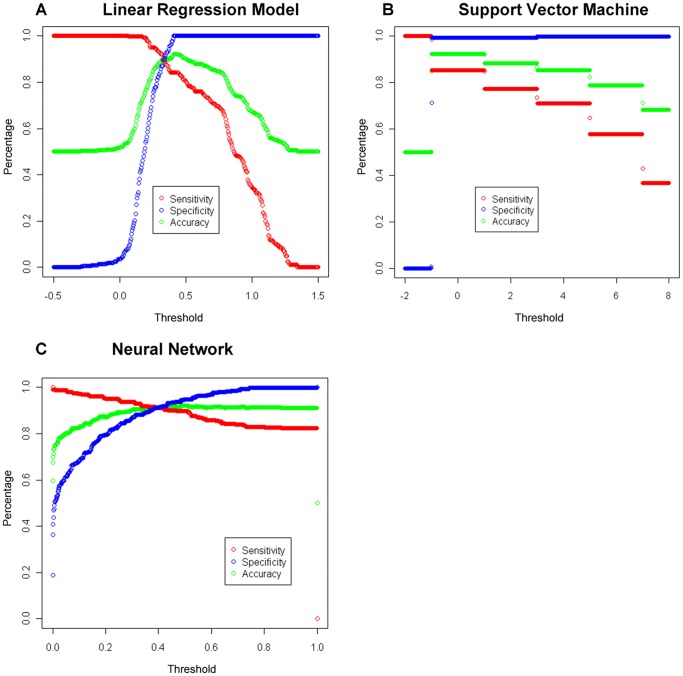
Dependence of minimotif multi-filter performance on threshold values for the linear regression and neural network models. Sensitivity, specificity, and accuracy for the linear regression (**A**) support vector machine (**B**) and neural network (**C**) models. Thresholds were selected by picking the best model in the 5-fold cross validation (model 2 of the linear regression and model 3 of the neural network) evaluated using the test dataset.

We next optimized the multi-filters. We repeated the minimotif filtering varying the filter score threshold to identify the maximum AUC for the best cross validation test in each of the three models (#2 of linear regression, #3 of support vector machine, and #3 of neural network). Plots showing the dependency of sensitivity, specificity, and accuracy on the filter threshold are shown for the linear regression, support vector machine, and neural network models in **Fig. 2**. The threshold dependence was typical of that for any filter. For these models, as the threshold increases, the sensitivity decreases as one would expect. As the threshold value increased the specificity for both models increased. The accuracy increased to a maximum and then decreased as the sensitivity dropped.

The plots shown in **Fig.2** were used to identify several threshold values for each model to help us select the best minimotif-filtering model. A threshold with the maximum accuracy is defined as the optimal threshold (T_o_). A stringent threshold that minimizes the number of false positives while retaining a high sensitivity is denoted as T_s_. The optimal threshold for the three minimotif filtering models produced accuracies above 90% with ∼ 85% true positive rate and less than 1% false positives (6% for the neural network) (**Table 3**). The stringent threshold produced less than 1% or in some cases no false positives (linear regression in **Table 3**), while retaining more than ∼90% of the true positives for the linear regression and neural network models (84% for the support vector machine model). Our evaluation of the selected models was also supported by the Matthews Correlation Coefficient (MCC) with a good performance of the filter combinations (MCC of 1 indicates a perfect prediction while 0 indicates no better than random).

**Table 3:** Summary of filtering statistics for three models.

Model	1Threshold	Sensitivity	Specificity	Accuracy	2MCC
Linearregression	T_o_ = 0.48	84.3%	100.0%	92.1%	0.85
	T_s_ = 0.48	84.3%	100.0%	92.1%	0.85
support vectormachine	T_o_ = −0.99	85.3%	99.3%	92.3%	0.85
	T_s_ = 3.00	73.5%	99.8%	86.6%	0.76
neuralnetwork	T_o_ = 0.50	89.8%	94.8%	92.3%	0.85
	T_s_ = 0.74	83.0%	99.8%	91.4%	0.84

1 T_o_: the optimal threshold with maximum accuracy; T_s_: the stringent threshold that minimizes the number of false positives while retaining high sensitivity.

2 MCC: Matthews Correlation coefficient.

Remarkably, the linear regression model with the Ts threshold produced 84% true positives with no false positives (**Table 3**), and the neural network model produced 83% true positives with less than 0.3% false positives. The ROC analysis further validated the optimized multi-filter approach as being far superior to any one filter by itself (**Fig. 1**). These ROC plots showed that each multi-filter model significantly outperformed any single filter by itself with AUCs above 0.95, whereas the AUCs for individual filters ranged form 0.72–0.88. The neural network had an AUC of 0.998 indicating that it is a superior filter model. This AUC was significantly better than that of the linear regression and the support vector machine models. The identification of highly efficient and accurate minimotif filter approaches represents an important milestone in the prediction of minimotifs.

In most minimotif searches the number of true positives far outweighs the negatives. Therefore, we also repeated the training and testing analysis on a larger data set where the negative data size was increased to 5-fold (10,000 randomly generated negative data points). This analysis for the combined filters showed a modest increase in the AUC and accuracy for all three algorithm models further supporting this approach for minimotif identity.

Since some of the individual filters had non-significant P values (**Fig. 1**), we questioned whether all five minimotif filters were needed to achieve the high level of accuracy. We repeated the filter analysis to find the best performing of all the five 4-combinations for each model. The average value of the AUCs and standard deviation (STD) of the 5-fold cross validation were calculated and a t-test was used to test which filters were optimal (P<0.05; **Table 4**). When the t-test identified more than one filter with similar performance, we reported the filter with highest average AUC, but also list the alternative filter combinations. The same approach was used to successively identify the best three-filter, and two filter combinations (**Table 4**).

**Table 4:** Variations of multi-filter combinations for each model.

				T_o_		T_s_	
linear regression	Combinations	AVG(AUC)	STD	Accuracy	MCC	Accuracy	MCC
5	CF+MF+FS+PPI+SP	95.5%	0.00	92.7%	0.76	90.1%	0.50
14	MF+FS+PPI+SP	95.4%	0.01	92.7%	0.75	90.2%	0.51
3	MF+FS+PPI	95.6%	0.00	92.9%	0.74	91.0%	0.55
2	FS+PPI	97.7%	0.01	95.5%	0.81	90.0%	0.50
support vector machine							
5	CF+MF+FS+PPI+SP	90.2%	0.08	96.6%	0.85	92.6%	0.62
14	MF+FS+PPI+SP	92.5%	0.04	97.2%	0.88	90.0%	0.50
13	FS+PPI+SP	92.4%	0.04	95.8%	0.82	92.5%	0.62
2	FS+SP	97.1%	0.02	94.6%	0.77	93.6%	0.67
neural network							
5	CF+MF+FS+PPI+SP	97.6%	0.04	97.2%	0.90	95.6%	0.79
4	MF+FS+PPI+SP	99.5%	0.01	96.4%	0.86	95.0%	0.76
13	FS+PPI+SP	99.1%	0.01	95.8%	0.82	91.7%	0.58
2	FS+PPI	97.8%	0.00	95.8%	0.79	90.4%	0.52

1 Alternative filter combinations that were not significantly different than the combination tested in the same row (P<0.05) were found: CF+MF+FS+SP for 4-filter combination in linear regression; CF+MF+FS+SP or CF+MF+PPI+SP for 4-filter combination, and MF+FS+PPI or MF+FS+SP for 3-filter combination in support vector machine; MF+FS+PPI for 3-filter combination in neural network.

To identify the best performing filters, the t-tests were also used to compare two-, three-, four- and five-filter combinations based on AUCs. One of the best filter combinations was the neural network model with the MF+FS+PPI+SP filters, having an AUC of 99.5%. This combination had an accuracy of 96.4% on the optimal threshold and an accuracy of 95.0% on the stringent threshold. For the linear regression the FS+PPI two-filter combination was significantly better than the other filter combinations. For the support vector machine, FS+SP was significantly better than FS+PPI+SP and CF+MF+FS+PPI+SP. For the neural network the MF+FS+PPI+SP and FS+PPI+SP were significantly better than the other filters. Collectively, this analysis identified the best model and filter combinations for increasing the accuracy of minimotif predictions.

### Implementation

We have now implemented multi-filtering on the Minimotif Miner website to help eliminate false-positive predictions (http://mnm.engr.uconn.edu and http://minimotifminer.org). The minimotif results table now lists the predictions ranked with the five-filter linear regression multi-filter score. We chose this model over the linear regression because so few false positives were produced while maintaining a very similar accuracy to the neural network. We chose the five-filter combination because, even though it had only a non-significant increase in AUC over some two-, three- and four-filter combinations, we could identify a threshold that had high accuracy with false-positives and a high percentage of true-positives. Those minimotifs with a score larger than 0.48 (a threshold above which only true positives surpass, and maximum accuracy of 92.1% is reached) are colored green, a score below 0.33 (which is the intersection of sensitivity and specificity shown in **Fig. 2A**) are colored red, and those between 0.48 and 0.33 are colored yellow. Those minimotifs where information is lacking and hence no score can be calculated are also colored red. A test of 20 randomly selected queries shows on average that 83% of minimotif predictions are rejected when using the threshold of 0.33 and 88% are rejected when 0.48 is used. This demonstrates that the trained filter successfully reduces the number of candidate minimotifs and the analysis of the global test set shows that most of the removed minimotifs are likely false-positives.

## Discussion

Minimotifs, by their definition are short, thus are of low complexity and highly prone to prediction of false positives, which limits their usefulness. As a result, tools that predict new minimotifs have developed scoring techniques or filter approaches. Even though a number of such scoring mechanisms are known, their effectiveness in reducing false positive rate has been limited [3], [7]–[9], [12], [13], [16]. One approach has been to try to increase the expected value by reducing the search space [17]. Most other approaches use different types of information to eliminate false positives. In our prior work we have considered pairwise combinations of select filters and found better filtering efficiency [13].

In this paper, we have developed and tested a new approach by combining multiple filters in an appropriate manner to achieve more effective filtering. An important decision to make in this case is on how to create a composite score, from several other disparate scoring metrics. We take the general view that we can pose the combination problem as one of learning. In this paper we have investigated three important ones, namely, linear regression, neural networks, and the support vector machine. Neural networks have been employed to solve different learning problems in biology such as identifying tyrosine based sorting signals and nucleolar localization sequences [18], [19]. Likewise linear regression and support vector machines have also been fruitfully employed in examples such as DNA splice site prediction, predicting antifreeze proteins sequences, NAD+ binding sites, etc. [20]–[22]. The suitability of these techniques for a given application can only be decided empirically because these techniques do not easily render themselves to complexity analysis. For instance, even for simple neural networks, convergence proofs are hard to derive. Similarly, for support vector machines, the separation achievable between the hyperplanes not only depends on the application, but also the specific set of data points.

Our empirical results show the robustness of the multi-filter in eliminating false positives and reaching a high accuracy. Meanwhile, joining different knowledge from each individual filter, the multi-filter also has limitations. The multi-filter works only if all the information of each individual filter for a minimotif is known, or all individual filters give valid results. Missing related information for one individual filter or incomplete data will limit the effectiveness of the multi-filter. This is part of the rationale for choosing the five-filter combination over other combinations with fewer filters with similar levels of significance. Also, there is bound to be bias in the datasets used in this analysis – the true positives are those reported for well studied proteins – while it is acknowledged that a tiny portion of false negatives are introduced in our generation of the negative dataset. Despite these limitations, the combined score with its excellent accuracy achievement represents the state-of-the-art in minimotif prediction and will be of great importance for biologists investigating proteins and disease mechanisms.

## Materials and Methods

### Data Sources

In order to both train and evaluate the multi-filter, it was necessary to compare a dataset of verified minimotifs with one containing known negatives. A set of ∼ 5,300 verified minimotifs exist in the MnM 2 database [8]. However, due to the nature of the individual filtering mechanisms, not all filters give definite results for each minimotif (for instance, minimotifs in which either the target or source proteins are undefined). The inclusion of such instances would bias the training towards those filters, which can act on incomplete definitions. Thus, the verified dataset was pruned to the 2,000 minimotifs that had unique source proteins, for which each filter yields a definite result, termed the “validated positive dataset”.

Since some minimotif sources proteins in the Minimotif Miner database have more than one target, we wanted to ensure that this was not providing a strong bias to our minimotif filtering analyses. 100 minimotif source proteins were randomly selected and pairwise alignment to all other minimotif source proteins in the dataset was performed using BLAST [23]. Approximately 10% of the source sequences had a bit score >30. Since this is often considered a threshold for common ancestry, this analysis indicates that there is some sequence similarity in the dataset used, but not enough to impact our conclusions.

Unfortunately, no database of verified negative minimotifs exists. Therefore, a negative dataset was computationally generated as previously described for our analyses of the PPI, CF and MF filters [12], [13]. First, pairs of (source protein, target protein) were randomly generated including no duplicates. For each source protein, minimotifs were found based on sequence matching from minimotifs in MnM database. In this manner, unique tuples of (source protein, minimotif, target protein) were generated. We created two data sets, one with the same number of data points as used in the positive dataset and one with a 5-fold excess of negative data points. These entries were treated as negative dataset and were estimated to have a negligible number of false negatives, which we expect would have negligible impact on the conclusions of our paper.

### Linear Regression

In linear regression, it is assumed that the relationship between a dependent variable and the associated independent variables is approximately linear and the model postulates the formula in (eq. 1).
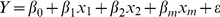
(1)Given 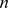 statistical observations of 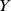 and 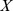, the linear regression problem is to find 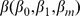 such that the linear model best predicts 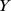 from 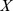, for example, to minimize 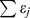 in the least square approach.

In the filter combination we envision that the independent variables are the outputs of the PPI filter (PPI), cellular function filter (CF), molecular function filter (MF), frequency score filter (FS), and the surface prediction filter (SP). The value of the dependent variable, called Score, is 1 or 0 and is decided based on whether the training entry is from positive data or negative data, respectively. Thus, the combination model is shown in formula (eq. 2).
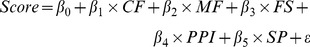
(2)The outputs of the cellular function filter and the molecular function filter are not binary. These filters output the shortest distance, or the least number of edges between cellular or molecular functions associated with the source and the target proteins in this training phase. Similarly, the frequency score filter outputs the number of minimotif occurrences divided by the length of the source protein. The surface filter outputs the likelihood that a motif is on the surface of the protein. The linear regression model was trained to get the parameter values of 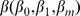 and then evaluated on the test data.

### Support Vector Machine

Support vector machine is a training and learning technique to classify data of different classes. In contrast to linear regression, which looks for a hyperplane crossing as many data points as possible, using the support vector machine produces a separating hyperplane which maximizes the margin between the closest points of two classes of data. That is, given 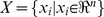 and 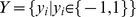 where 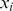 is a data point in *n*-dimensional space and 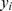 is the class to which 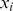 belongs, the algorithm identifies a hyperplane 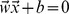 to maximize the distance between two parallel hyperplanes (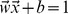 and 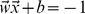) which separate the data points into two groups. The distance between those two hyperplanes is 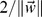. Therefore, the support vector machine tries to find a hyperplane 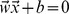 to minimize 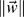, given 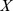 and 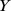.

The original support vector machine is a linear classification technique [24]. With a kernel function, the non-linear support vector machine can be created to get a curve with the maximum margin [25]. Non-linear separation is achieved by transforming the data points from the original space into a new space in which they can be more easily separated, which is done by replacing the linear dot product operations for vectors with (non-linear) kernel functions. Several kernel functions can be used in the support vector machine, like polynomial, radial basis function, and sigmoid. A linear kernel also exists to recover the computation back to the linear support vector machine. In the proposed method, we used the original linear support vector machine model based on the assumption of the independence of individual filters.

The training data for the support vector machine was collected as in the linear regression, except that the parameter of score of positive and negative data is not necessary here. Assuming a high dimensional space, in which each dimension indicates a filter, given a motif with its output values of all filters, this motif is located at the coordinate of its filters’ output, like (PPI, CF, MF, FS, SP). Support vector machine is designed to construct a hyperplane to separate the motif points of positive data from those of negative data in the training phase and such a hyperplane is tested in the evaluation.

### Neural Network

Neural network, or artificial neural network, is a model to simulate biological neural networks. Neurons are the basic units or nodes in this network, which are interconnected layer by layer. Each neuron is connected to neurons in adjacent layers based on the edge weights. Each neuron works independently and accepts inputs (or input signals) from the previous layer. Layer by layer, all the neurons are combined together for a final output, to model the relationship between the inputs and their desired output. Sometimes an activation function defines whether to activate a neuron by thresholding its input values.

Mathematically, the neural network uses a function 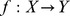, in which each neuron contributes to the final output based upon edge weights. The output of the 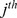 neuron on the 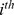 layer of a neural network is a function 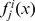 that is based on the outputs from the 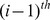 layer 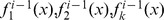. In particular, 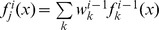. The output of the 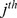 neuron on the first layer is defined as 
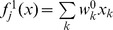
, where 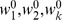 are the weights on the inputs 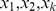.

In the filter combination we have constructed, the outputs of individual filters (PPI, CF, MF, FS, SP) are used as input data 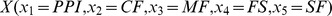 and for 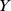 we use 1 for positive data and 0 for negative data. By training this model, it is expected that the filter’s output for positive data will be ∼1, while ∼ 0 for negative data. When training and testing the neural network model, hidden layers were eliminated as much as possible without sacrificing performance. The reported neural network has two hidden layers.

### Cross Validation

A 5-fold cross-validation was used to validate linear regression, support vector machine, and neural network models as follows: 1) partition the positive and negative data into five equally sized groups: 400 positive and 400 negative data points; 2) for each group, leave one group out and use the remaining data to train a linear model and test it with the left-out group of data; 3) Repeat training and testing five times till each group is used as testing data once. Evaluation of the filters with Receiver Operator Curve (ROC) was performed. A threshold is used to determine whether a new query (source, motif, target) should be retained or eliminated by the multi-filter. The optimal threshold is determined recursively for a maximum accuracy (eq. 3, TP: true positive; TN: true negative; FP: false positive; FN: false negative; P: positive data; N: negative data).
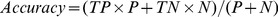
(3)The Matthews Correlation Coefficient (MCC) is used to show the performance of the multi-filter (eq. 4). The value is perfect if it is 1, and 0 means not better than a random prediction.
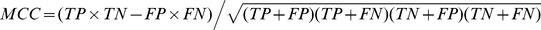
(4)

